# Observations on the Membranes

**Published:** 1801-11

**Authors:** 


### *Observations on the Membranes. By* Cit. Bichat.

THE membranes may be divided into two claffes, the fimple and compound membranes the firft of which are endued; with a peculiar organization, and the latter confifts of two or three fimple membranes. The firft clafs has three fpecies, viz.

1. *Mucous membranes*, fecerning mucus by fmall glands, and covering the inner furface of all hollow organs that communicate with the furface of the body; of this kind are the membranes of the nofe, the mouth, œfophagus, urinary bladder, uterus, and of all excretory canals.

2. *Serous membranes*, exhaling a lymphatic liquor from the blood, fuch as the pericardium, pleura, peritonæum, the membranæ

branæ vaginales, the membrana arachnoidea, the membranes of the joints, and the vagina tendinum.

3. *Fibrous membranes*, consisting of fibres like the tendines, but secerning nothing, the periosteum, dura meninx, sclerotica, aponeuroses, &c. belong to them.

From these three species of membranes are composed the *fibro-serous*, the *sero-mucous*, and the *fibro-mucous* membranes, besides which there are some others of an unknown or peculiar organization, and, last, the membranes formed by diseases.

All mucous membranes may be considered as parts extended on two general surfaces, the first of which begins in the mouth, the nose, and the eye; the mucous membrane covers the cavity of the mouth and the nose, whence it is continued in the excretory canals of the glands of the ears and cheeks; and having formed the conjunctiva, it goes down to the puncta lacrymalia, the saccus lacrymalis, and the canalis nasalis; from hence it continues to the cavities of the nose, and slips down the throat, and having given a part to the tuba Eustachii, with which it penetrates into the internal ear, it proceeds to the windpipe and its branches, to the œsophagus and stomach, where it is propagated to the duodenum, and having left two processus, one for the ductus coledochus and hepatica, and another for the canal of the pancreas, it terminates in the anus, where it passes over to the skin.

The second expansion of the membrana mucosa begins in the urethra of the male sex, whence it partly proceeds to the bladder, the ureteres, the kidnies, and their particles and canals, partly continues in the excretory canals of the prostata, the ductus ejaculatorii, the vesicula seminalis, the vas deferens, &c. In the female sex it commences in the vulva, and penetrates partly to the urinary ways like as in men, partly it goes up to the vagina, the uterus, and tubes, at the opening of which it gets in contact with the peritonæum.

The mucous membranes differ in their internal organization from the fibrous and serous ones, by consisting of several lamellulæ, similar to those of which the skin is composed, viz. the *epidermis*, the *corpus papillare*, and the *corium*.

The epidermis may be separated by hot water from the other lamellulæ on the tongue, the palate, and the throat; but deeper down it is very difficult to show it, as Mr. B. never succeeded in separating it from the inner membrane of the stomach and intestines. It has, like the common epidermis, a tendency of becoming callous by pressure. Mr. B. was not able to discover a *rete mucosum* under it, as in the common epidermis.

The

The corpus papillare of the mucous membranes is eafily to be diftinguifhed, particularly at their origin, though it appears lefs evident deeper downwards; the membrana flocculofa however feems to be nothing but this corpus papillare, which chiefly confifts of nerves, to which it owes its fenfibility. The corium of the mucous membranes confifts of feveral ftrata of condenfated tela cellulofa; it is ftrong in the palate, tongue, and nofe, but of a finer texture in the ftomach and inteftines, and almoft not to be perceived in the urinary bladder, and the veficula fellea. The mucous membranes are endued with a great quantity of mucous glands, fituated either in the corium or under it; and likewife with many fuperficial veffels, on account of which it is expofed to frequent hæmorrhagies. The ftructure of thefe membranes differs according to the regions in which they are found, and, by the fame reafon, they differ alfo in the degree of irritability which they enjoy; whereas the ferous membranes have every where the fame appearance and degree of irritability.

With regard to the difeafes of the mucous membranes, the following queftions may be propofed for being farther difcuffed. Why do thefe membranes never grow together, when they are inflamed, which always happens to the ferous membranes? Why does the fecretion increafe in inflamed mucous membranes, while the ferous membranes become almoft perfectly dry in a fimilar ftate? Why do polypi, which are never obferved but in mucous membranes, moftly appear at the origin of thefe membranes, and in the neighbourhood of the fkin?

The ferous membranes cover outwardly the greateft part of thofe organs which have mucous membranes inwardly, and are particularly obferved in parts expofed to ftrong motion and friction. They alfo furround all organs neceffary for life, as the brain, the heart, the lungs, all the inteftines, the tefticles, &c. They are not combined with each other, but fingle, and their extent taken together is greater than that of the mucous membranes and of the fkin.

The ferous membranes are of two kinds; to the firft of which, the pleura, pericardium, peritonæum, the membrana arachnoidea, and the tunica vaginalis belong; the fecond comprifes the capfulæ of the vaginæ tendinum, otherwife called burfæ mucofae, or capfulæ fynoviales, and the fynovial membranes. The liquors fecerned by both kinds of membranes, differ in fome refpects. Dropfies do not fo frequently occur in the fynovial membranes at the fame time with fimilar affections of the membranes in great cavities.

The external furface of the ferous membranes adheres every where to the neighbouring parts, which combination with their refpective respective organs differs from that of the fibrous membranes, the latter being so intimately connected with the organs by vessels, that the part to which they adhere dies when they are taken away; for instance, a bone when deprived of its periostium. The serous membranes however, are, as it were, heterogeneous parts to the organs, to which they adhere, and their life is independent of the life of the organs which are surrounded by them. The serous membranes are of a white and somewhat glossy colour, of different thickness, and transparent, and consist only of one stratum of tela cellulosa, upon which blisters have not the least effect, as Mr. B. frequently observed, on applying them to the naked intestines. As they chiefly consist of absorbent vessels, they may be considered as great reservoirs, situated in the middle between the absorbing and exhaling system, into which the lympha runs over from one system, and in which it undergoes some changes before it is imbibed by the other system. Blood vessels seem not to be essential to these membranes. Respecting the diseases to which the serous membranes are exposed, Mr. B. proposes the following queries:— Why are the upper membranes, the pleura, the pericardium, but particularly the arachnoidea, more rarely affected by dropsies than the lower ones, as the peritonæum, tunica vaginalis, testiculi, &c.? And why do the synovial membranes not share in dropsical affections with the tela cellulosa and other serous membranes? In what relation are the purulent and viscous exsudations in inflamed serous membranes with the increased secretion in the mucous membranes, which generally takes place at the same time, &c.?

The fibrous membranes are observed to cover several organs, as the bones, the eyes, the testicles, the penis, and the kidneys, and we find them also in the interstices of muscles, about the joints, &c. They seem to be all united with the periostium, except the perichondrium of the cartilages of the larynx, the tunica albuginea, and the membrane of the kidneys. It constitutes a character of these membranes, that they never form any plaits, but are exactly adapted to the form and size of the parts which they surround, except the dura meninx.

The fibrous membranes may be comprehended under two classes; to the first of which belong, 1. The aponeuroses, either serving as covers to the organs or for insertion. 2. The fibrous capsulæ of the joints. 3. The fibrous vaginæ of the tendines. To the second classes, the periostium, the dura meninx, the sclerotica albuginea, &c. may be reckoned. The membranes of this class differ from those of the other, by being immediately united to the organs which they cover, whereas the first seem

ſeem to be heterogenous to the parts with which they are combined, poſſeſſing an independent organization and life.

The organiſation of the fibrous membranes is every where the ſame, as they only conſiſt of one lamina, with exception of the dura mater, which has two plates, where it forms its cavities. The chief organic element of the fibrous membranes is a peculiar, ſolid, elaſtic, inſenſible fibre, of little contractility, which cannot be diſſolved into tela celluloſa by maceration. Of this fibre the tendons and ligaments are likewiſe formed, which only differ from the fibrous membranes by the fibres of thoſe parts being collected in parallel bundles, now and then croſſing one another, whereas they are formed here by a reticular texture. Theſe membranes are every where penetrated by blood veſſels, which conſtitute an eſſential point of their organiſation.

By theſe ſimple membranes uniting, three kinds of compound membranes are formed.

1. *The fibro-serous membranes* ariſing from the union of the fibrous and ſerous membranes, which naturally have a great tendency of uniting with each other. This is particularly the caſe, 1, with the *arachnoidea*, being intimately connected with the internal ſurface of the dura meninx; 2, with the *albuginea*, which receives a lamina from the membrana vaginalis; 3, with the greateſt part of the pericardium, that is inſide of a ſerous, outſide of a fibrous texture; 4, with all the *synovial membranes*, which partly unite with the capſulæ articulares, partly with the ſheaths of the tendons. In general, this compoſition of membranes will be only met with where the organs are not expoſed to conſiderable changes in their ſize, and it would by no means be adapted for parts whoſe ſize is changeable, as, for inſtance, the ſtomach, urinary bladder, uterus, &c.

2. *The sero-mucous membranes.* There are very few inſtances of ſuch a combination in animal organization, at leaſt both membranes ſeldom come in immediate contact with each other, where they are united for the formation of any organ, being moſtly (for example in the inteſtinal canal) ſeparated by a ſtratum of muſcular fibres. The loweſt part of the veſica fellea, however, is an inſtance of their immediate combination; but it is not ſo intimate, that they might not be conſidered as ſeparate membranes.

3. *The fibro-mucous membranes* are met with, 1, In the *ureters* ariſing from the membrane of the kidneys and the mucous membrane of the urinary bladder. 2. In the *vas deferens*, 3. In the *membranous part* of the *urethra*. 4. *The membrana nasalis* is alſo moſt probably compoſed of perioſtium and of a mucous membrane. 5. In the internal coverings of the *ear*, and in the *tubæ Fallopii*, a ſimilar union of both membranes moſt likely

 takes takes place, which is always ſo intimate, as not by any means to be ſeparated.

Beſides the above membranes, we find ſome which are either of a particular ſtructure, or have not yet been exactly examined, and therefore cannot well be ranked among the former, namely, the *membrana intermedia* of the arteries, the *membrana interna* of the veſſels, the membrane which covers the inſide of the cylindrical bones, the *iris* and *choroidea*, the *retina* and *pia mater* or *meninx*.

Mr. *Bichat* calls abnormous or irregular membranes, thoſe of which the bags of the Hydrops ſaccatus or cyſticus, Hydatides, Steatomata, Atheromata, &c. and the cicatrix are formed. The bags which ariſe in thoſe diſeaſes are of a ſerous texture, and ſeem not merely to be owing to an extenſion and compreſſion of the tela cellulofa.

The ſynovial membranes exhale a liquor, called ſynovia, which is in many reſpects ſimilar to that ſecreted by the ſerous membranes, with which they have indeed a great analogy in their internal organization, as they likewiſe conſiſt of tela celluloſa.

